# Polymorphic light eruption shows aberrant expression of epidermal tight junction proteins in unexposed and UVR-exposed skin: an experimental study

**DOI:** 10.1007/s43630-025-00845-1

**Published:** 2026-01-19

**Authors:** Emma Pond, Lesley E. Rhodes, Craig Johnson, Neil K. Gibbs, Catherine A. O’Neill

**Affiliations:** 1 Centre for Dermatology Research, Faculty of Biology, Medicine and Health, NIHR Manchester Biomedical Research Centre, University of Manchester, Manchester, UK; 2 Photobiology Unit, Manchester Academic Health Science Centre, Salford Royal Hospital, NCA NHS Foundation Trust, Greater Manchester, UK; 3 Present Address: Curapel, Stuart House, Chepstow, Wales

**Keywords:** Polymorphic light eruption, Tight junction protein, Claudin-1, Skin barrier, Photoantigen

## Abstract

Polymorphic light eruption (PLE) is a common photodermatosis which is believed to be attributable to an immune reaction to an unidentified photoantigen. The epidermal barrier deters skin entry of photoantigens, but its potential contribution to PLE pathogenesis is unexplored. Our objective was to examine the expression of key barrier proteins in PLE patients in vivo compared with healthy controls, both at baseline and after ultraviolet radiation (UVR) exposure. Seven PLE patients and 10 healthy volunteers were recruited. Small areas of buttock skin were exposed to 80 mJ/cm^2^ erythemally-weighted UVR (Philips TL12 broadband UVB source, 280–400 nm) and biopsies were taken at 24 h post-UVR and from unexposed buttock skin. Skin sections were assessed by immunostaining for expression of barrier proteins (claudins-1, -4, -7, -12; occludin, filaggrin, loricrin and involucrin), and the proliferation marker Ki67. It was demonstrated that unexposed skin from PLE patients had remarkably lower claudin-1 expression versus healthy volunteers (*P* < 0.001), while expression of Ki67 was significantly increased. UVR-exposure of healthy volunteer skin reduced claudin-1 expression at 24 h post-UVR (*P* < 0.01), while in contrast, UVR-exposure of PLE patients decreased the expression of claudin-7 and increased the expression of claudin-12 (both *p* < 0.05). Thus tight junction protein expression is altered in people with PLE, both in baseline skin and in their response to UVR exposure. These novel findings suggest a potential role for tight junction proteins in PLE pathogenesis and warrant further exploration.

**Graphical abstract:**

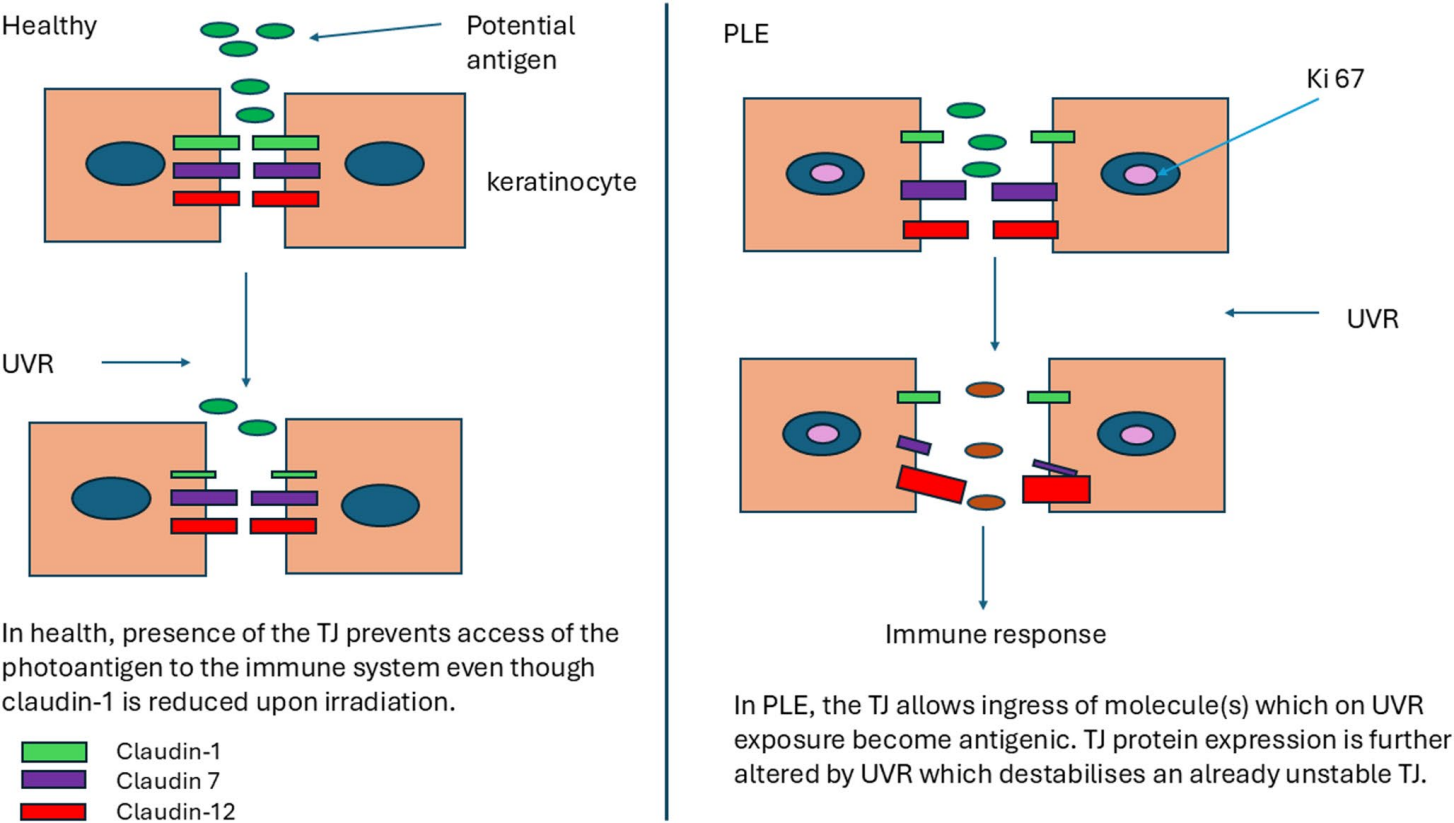

## Introduction

Polymorphic light eruption (PLE) is the most common photodermatosis, affecting around 18% of the European population1 [1]. The condition presents as a non-scarring, pruritic eruption of papules, vesicles and/or plaques, typically appearing on the skin hours following sun exposure [2]. PLE is grouped within the photodermatoses believed to be attributable to dysregulated immune responses to ultraviolet radiation (UVR) [3–8] while the potential role of the skin barrier has remained unexplored.

PLE is thought to be a delayed hypersensitivity response to an antigen produced following exposure to UVR [3, 4]. PLE patients have impaired UVR-induced immunosuppression, with a reduction in UVR-induced tolerance to contact allergens compared to healthy people [5–7]. Exposure to UVR induces an initial influx of CD4 + T lymphocytes followed by CD8 + T lymphocytes, associated with increased numbers of dermal dendritic cells, macrophages and Langerhans cells [reviewed in 8]. Recent research has implicated specific inflammatory proteins of the interleukin (IL)-1 and IL-36 family [9]. Patients with PLE also have low serum vitamin D status [10], which may contribute to immune abnormality in PLE [reviewed in 11]. The nature of the allergen is unknown but it is believed to be a cutaneous molecule rendered immunogenic by UVR. The high prevalence of PLE suggests the putative allergen is ubiquitous and may work in concert with other factors in susceptible individuals.

We considered that an aberrant barrier component may contribute to the aetiology of PLE, reasoning that if the allergen is ubiquitous, it would need to gain access to the immune system in order to induce PLE. A leaky skin barrier may be a key part of this process. The skin barrier comprises not only the stratum corneum, but also tight junctions (TJs) which are present in the granular layer of the epidermis [12]. TJs are multi-protein complexes that seal the pathway between adjacent epithelial cells thus preventing the free movement of molecules, ions and water across the epithelium [13–15]. Although their full role in the epidermis is incompletely understood, their presence is crucial for epidermal barrier function [16]. In skin, aberrant TJs have been associated with atopic dermatitis [17, 18], chronic plaque psoriasis [19, 20] and ichthyosis [21]. Furthermore, TJs, at least in rodent models and human keratinocytes, are regulated by UVR [22, 23].

In this novel study, we compared the structure of epidermal TJs in healthy volunteers and patients with PLE. We examined unexposed as well as UVR-exposed buttock skin to allow for investigation for differences at baseline. Detailed immunohistological examination of skin sections was performed for expression of the major skin TJ proteins claudins-1,−4,−7 and − 12 and occludin, all of which have been well characterised previously in both human and mouse skin [24, 25]. In addition, we assessed the stratum corneum markers filaggrin, loricin and involucrin and the proliferation marker Ki-67, alterations in which have been associated with changes in claudin expression [17]. Finally, we measured trans-epidermal water loss (TEWL) in healthy volunteers and patients with PLE both at baseline and in response to UVR.

## Methods

### Study participants

The ‘Skin Barrier in PLE’ study ran from January 2014 until September 2016. Ethical permission was granted by Greater Manchester North West Research Ethics Committee (Research Ethics Committee reference 13/NW/0797). The study was performed in accordance with the Declaration of Helsinki 1964 (revised Brazil 2013). All participants gave written informed consent. All clinical procedures were performed in the Photobiology Unit, Centre for Dermatology Research, Salford Royal Hospital, NCA NHS Foundation Trust, Salford, Greater Manchester, UK.

The study volunteers comprised 7 patients with moderately severe PLE (4 female and 3 male, age range 33–47 years, whose diagnosis was made by a photodermatologist after their investigation in the photodiagnostic service, Photobiology Unit, Centre for Dermatology, Salford Royal Hospital) and 10 healthy people (7 female and 3 male, age range 25–57 years). All volunteers were white Caucasian, sun-reactive skin types I-III.

### UVR exposure and skin sampling

The minimal erythemal dose (MED) of each subject was assessed using a series of ten doses of erythemally-weighted UVR (doses 7–80 mJ/cm^2^; TL-12 lamp, Philips, Amsterdam, The Netherlands) applied to the previously unexposed upper buttock skin (1 cm diameter circular sites). The highest dose of 80 mJ/cm^2^ was applied to 2 sites for purposes of skin biopsy, a dose equating to ~ 2.5xMED for study participants. The TL-12 unit emitted a spectrum of UVR between 280 and 400 nm (65% UVB; peaks 310 nm and 365 nm); irradiance was measured using a Waldmann meter (custom design, Professor Brian Diffey, Newcastle, UK) calibrated against a National Physical Laboratory (Teddington, UK) standard. The mean (SD) MED for all volunteers was 32 (10) mJ/cm^2^, with no significant difference between groups (not shown).

Twenty-four hours post-UVR, all participants had two 4 mm biopsies taken from the areas exposed to 80 mJ/cm^2^ UVR and two 4 mm biopsies from an unexposed area of upper buttock skin. One biopsy from each of the UVR-exposed and unexposed sites was snap-frozen for histological assessment and the other was used for assessment of TJ function.

### Measurement of transepidermal water loss

TEWL was measured in triplicate in volunteers using a Vapometer (Delfin Technologies, Finland) in a temperature and humidity-controlled environment.

### Analysis of biopsy samples

Biopsies were embedded in OCT mounting medium (Cell Path, Powys, UK), and 10 μm sections were cut and mounted onto glass slides. After air-drying at room temperature for 10 min sections were fixed (50:50 acetone: methanol for involucrin, loricrin and claudin isoforms, and 4% paraformaldehyde for occludin, filaggrin and Ki-67) then washed in 1X tris buffered saline (TBS). Sections were incubated for 1 h at room temperature in blocking solution (1% (w/v) BSA/10% (v/v) goat serum) then incubated with primary antibody (rabbit polyclonals anti-claudin 4,7,12, filaggrin, loricrin) and mouse monoclonal anti-occludin, involucrin, claudin-1 and Ki-67 (all from Invitrogen, Paisley, UK). Negative control tissue sections were covered with a drop of blocking solution containing no antibody. The slides were incubated overnight at 4 °C in a humidified chamber, washed in TBS for 15 min then labelled with Alexa Fluor 488 goat anti-rabbit or goat anti-mouse IgG (as appropriate) at room temperature for 45 min. Slides were washed in TBS and counterstained with 4’,6-diamidino-2-phenylindole (DAPI, Invitrogen, Paisley, UK) for 1 min. Slides were then mounted and images taken with a Keyence BZ-8100 microscope (8000 Series, Keyence, Japan) using the BZ observation software (Keyence, Japan). For all sections, the exposure time was set using the negative control slide and then kept constant for all other images captured.

### Image analysis

Three sections from each sample were used for all measurements. Images were analysed using ImageJ (National Institutes of Health, Bethesda, Maryland, USA). For pan-epidermal markers the freehand tool was used to draw around the epidermis and the percentage area of the epidermis that produced a signal was calculated. To investigate granular layer markers the line intensity tool in ImageJ (National Institutes of Health, Bethesda, Maryland, USA) was used. Measurements were taken from the start to the end point of the TJ protein expression. Plots of the profile of intensity were then generated to represent fluorescence intensity.

### Sample size and statistical analysis of data

The sample size was selected on the basis of feasibility of recruitment of volunteers to a multiple skin biopsy study, and appropriateness for an experimental study. Analysis of data was carried out using Microsoft Excel© (Microsoft Corporation, USA). Data are presented graphically as mean ± standard deviation (SD). After confirming that the data met appropriate assumptions (normality and homogeneity of variance) data were evaluated with the unpaired two-sided Student’s t-test or by one-way ANOVA followed by Bonferroni’s multiple comparison tests to determine statistical significance.

All statistical tests were carried out using Prism 6 (GraphPad Prism 6, GraphPad Software, Inc. La Jolla, CA 92037 USA). Differences were considered statistically significant at a P-value < 0.05.

## Results

### Characteristics of the unexposed epidermis of patients with PLE versus healthy controls

**Fig. 1:**
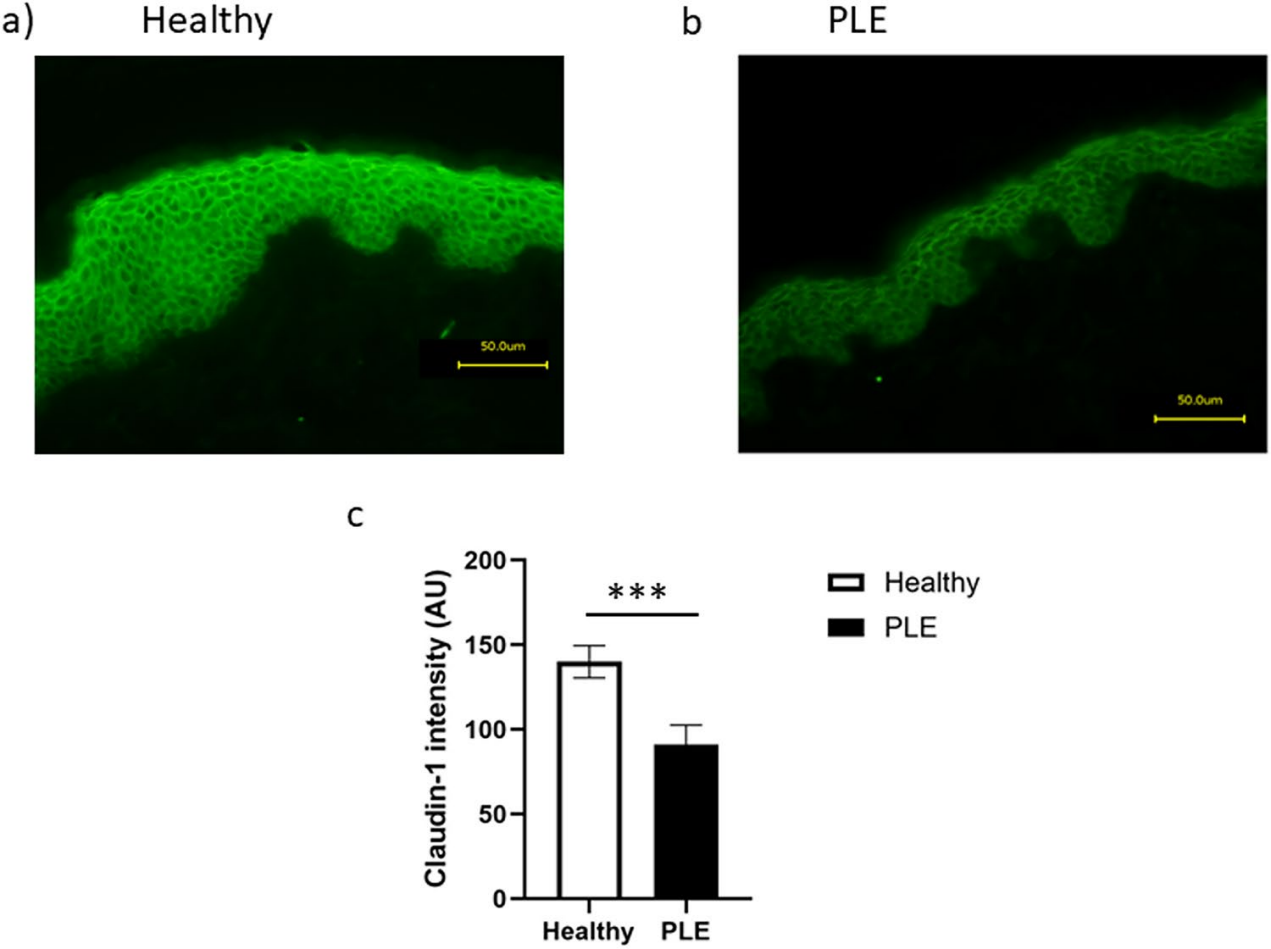
Claudin-1 staining is reduced in skin from patients with PLE. Claudin-1 staining in skin from **a** healthy volunteer and **b** patient with PLE. **c** Quantification of the signal intensity shows a significant reduction in claudin-1 staining in skin from patients with PLE (*n* = 7) compared to healthy volunteers (*n* = 10, *p* < 0.001). Data shown are mean ± SD

Skin from healthy volunteers and patients with PLE was stained for the TJ proteins, claudins − 1,−4, −7,- 12 and occludin and the stratum corneum markers, filaggrin, involucrin and loricrin. The staining patterns were compared between patient and healthy groups. In the epidermis of patients with PLE, the expression of claudin-1 was significantly less than in healthy controls (*p* < 0.001, Fig. 1a-c). By contrast, the expression of other barrier markers was identical in the skin of patients with PLE and healthy control skin (data not shown).

**Fig. 2:**
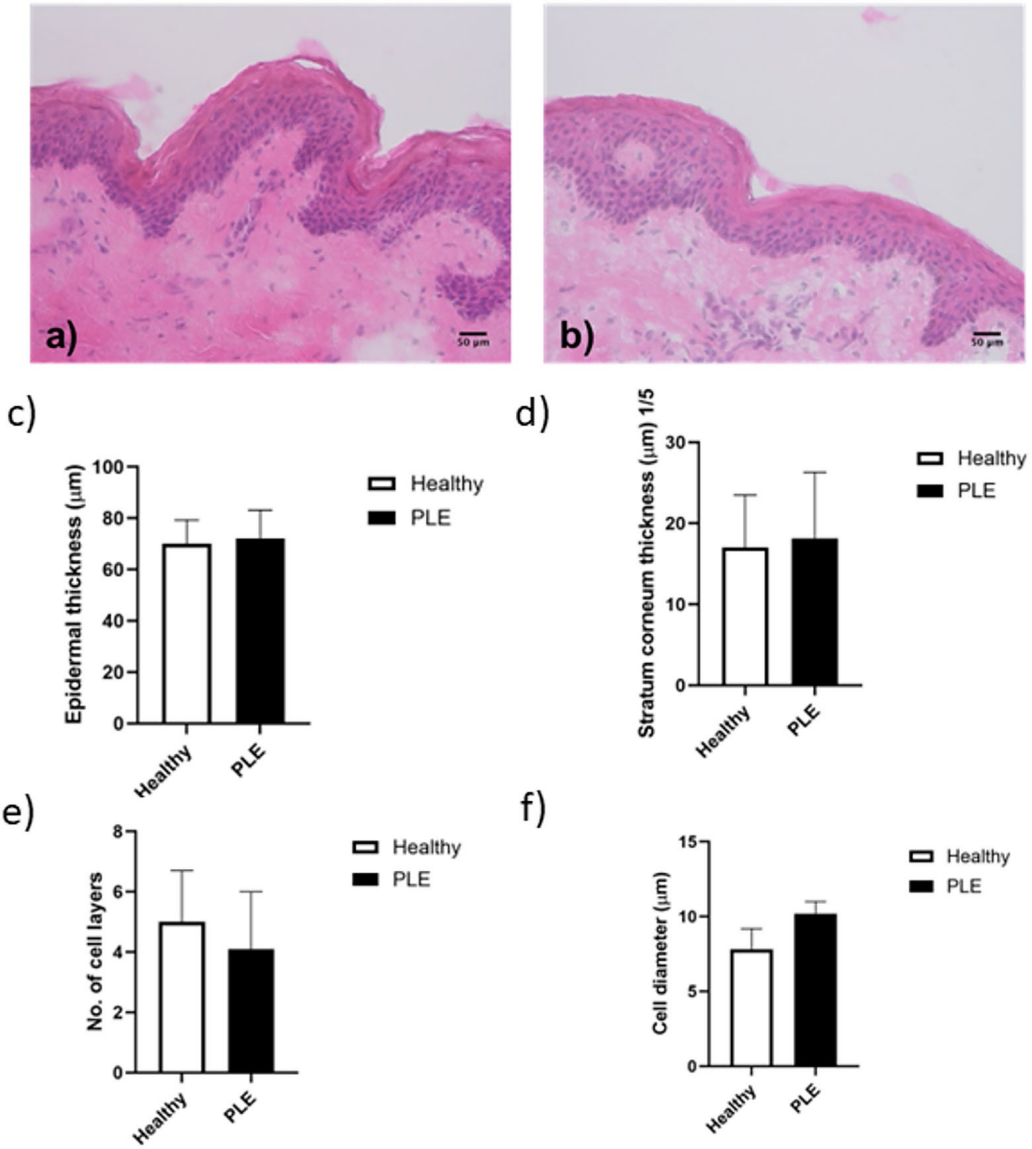
Epidermal morphology is unaltered in patients with PLE. Haemotoxylin and eosin staining of the skin of (**a**) healthy volunteers and (**b**) patients with PLE. Neither epidermal thickness (**c**), stratum corneum thickness (**d**) the number of cell layers (**e**) nor the cell diameter (**f**) was different between the two groups. Data shown are mean ± SD

**Fig. 3:**
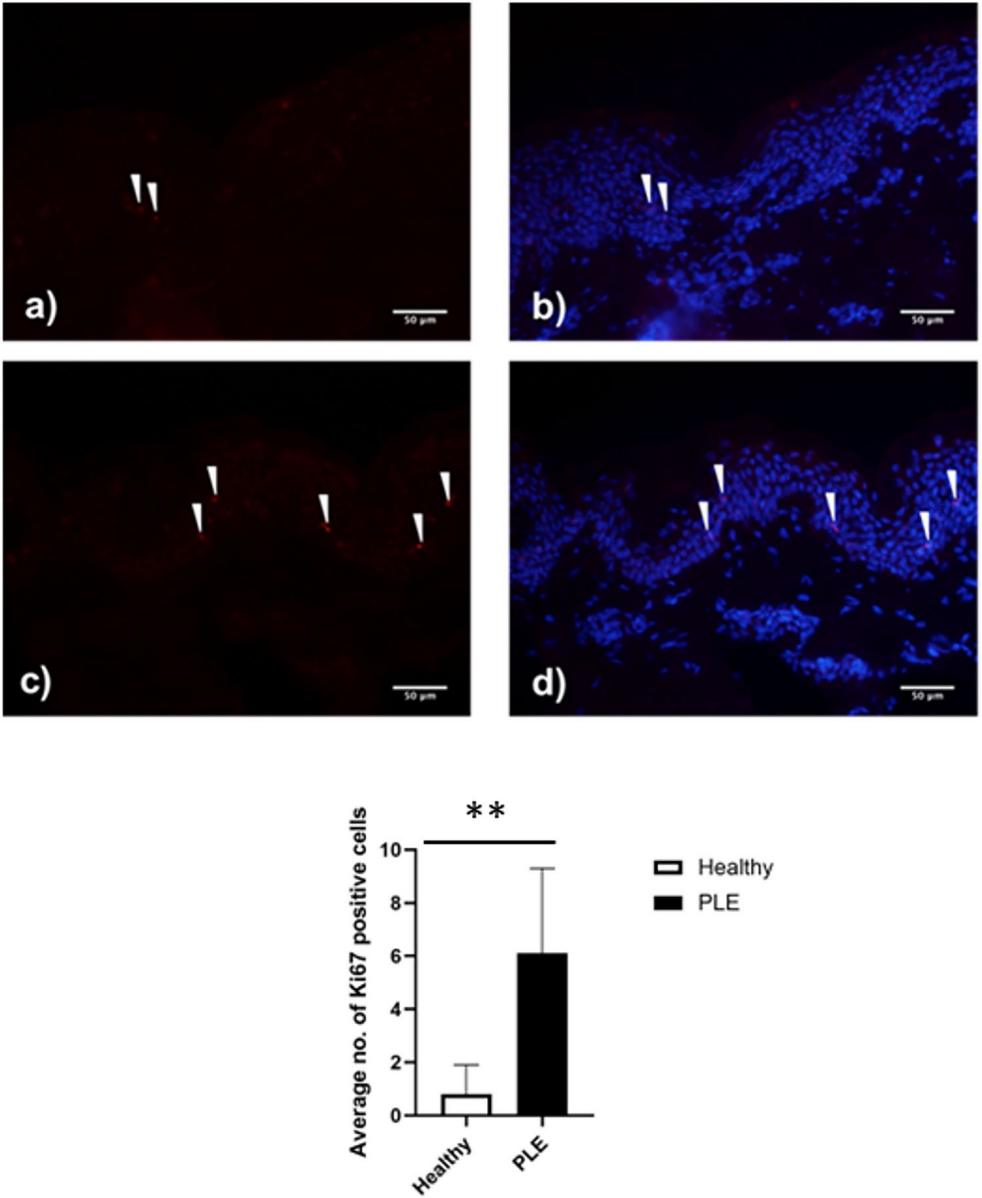
The epidermis of patients with PLE contains more proliferating keratinocytes than skin from healthy volunteers. Ki-67 staining of skin from healthy (**a**, **b**) and patients with PLE (**c**, **d**). Arrows denote KI-67 -positive cells (pink staining). The nuclei are counterstained with DAPI (blue staining). Quantification of the images shows a significant increase in Ki-67 positive cells in the skin of patients with PLE (*n* = 7) compared to that of healthy volunteers. (*n* = 10, *p* < 0.01). Data shown are mean ± SD

Since differences in claudin-1 staining could be attributed to changes to the morphology of PLE keratinocytes we investigated epidermal and stratum corneum thickness, number of cell layers and cell diameter in PLE patient skin versus healthy skin. No morphological differences were seen (Fig. 2a-f), while the skin of patients with PLE exhibited more cells that were positive for Ki-67 than that of healthy skin (Fig. 3a-e).

### Tight junction protein expression is perturbed by UVR in epidermis from healthy volunteers and patients with PLE

**Fig. 4:**
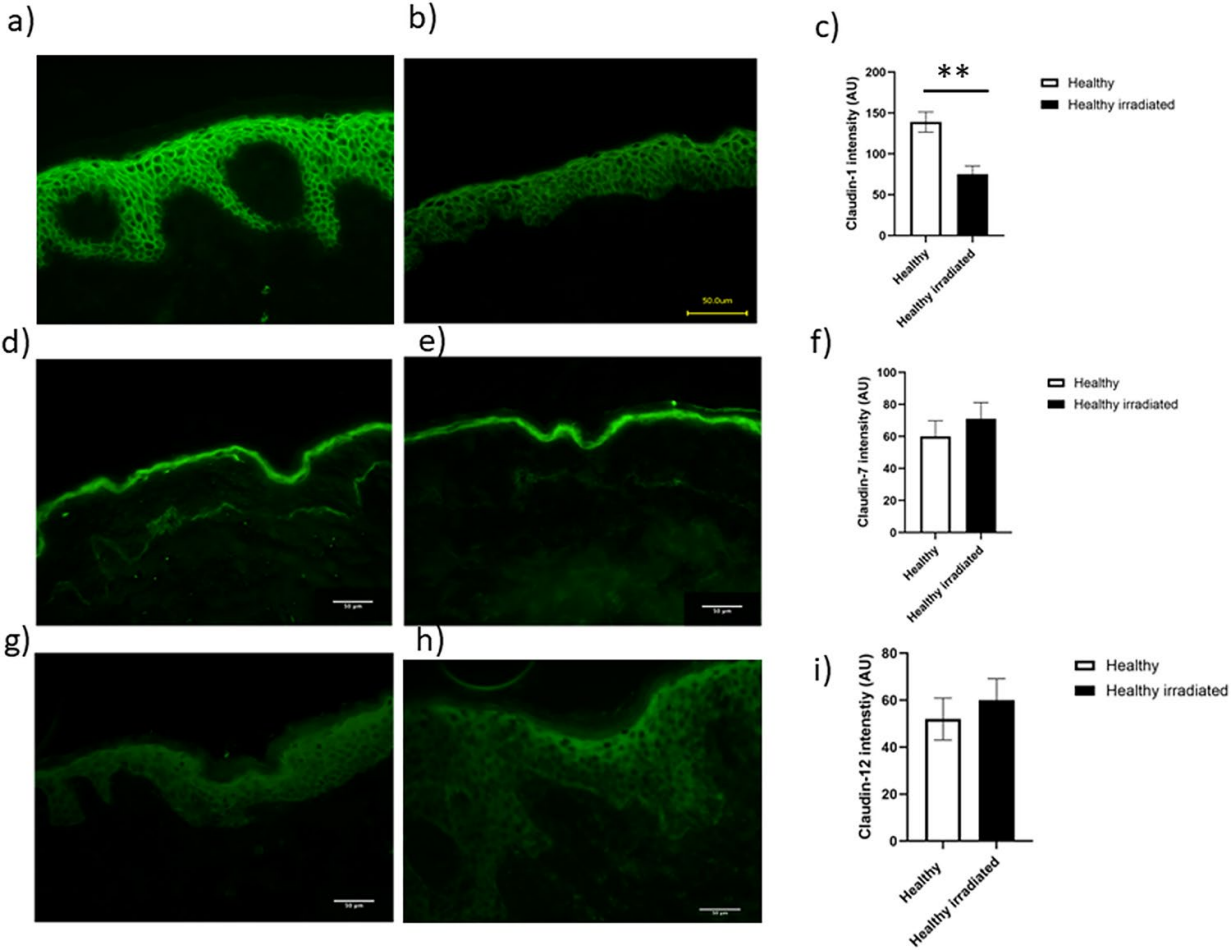
Claudin-1 staining is reduced in UVR-irradiated healthy volunteer skin. Healthy volunteers were exposed to a single dose of UVR and a biopsy taken 24 h post irradiation. Claudin-1 (**a**, **b**,**c**) staining was significantly reduced in the irradiated skin (*n* = 10, *p* < 0.01). There was no change in the quantity of staining for claudin-7 (**d**, **e**, **f**) or claudin-12 (**g**, **h**, **i**). Data shown are mean ± SD

**Fig. 5:**
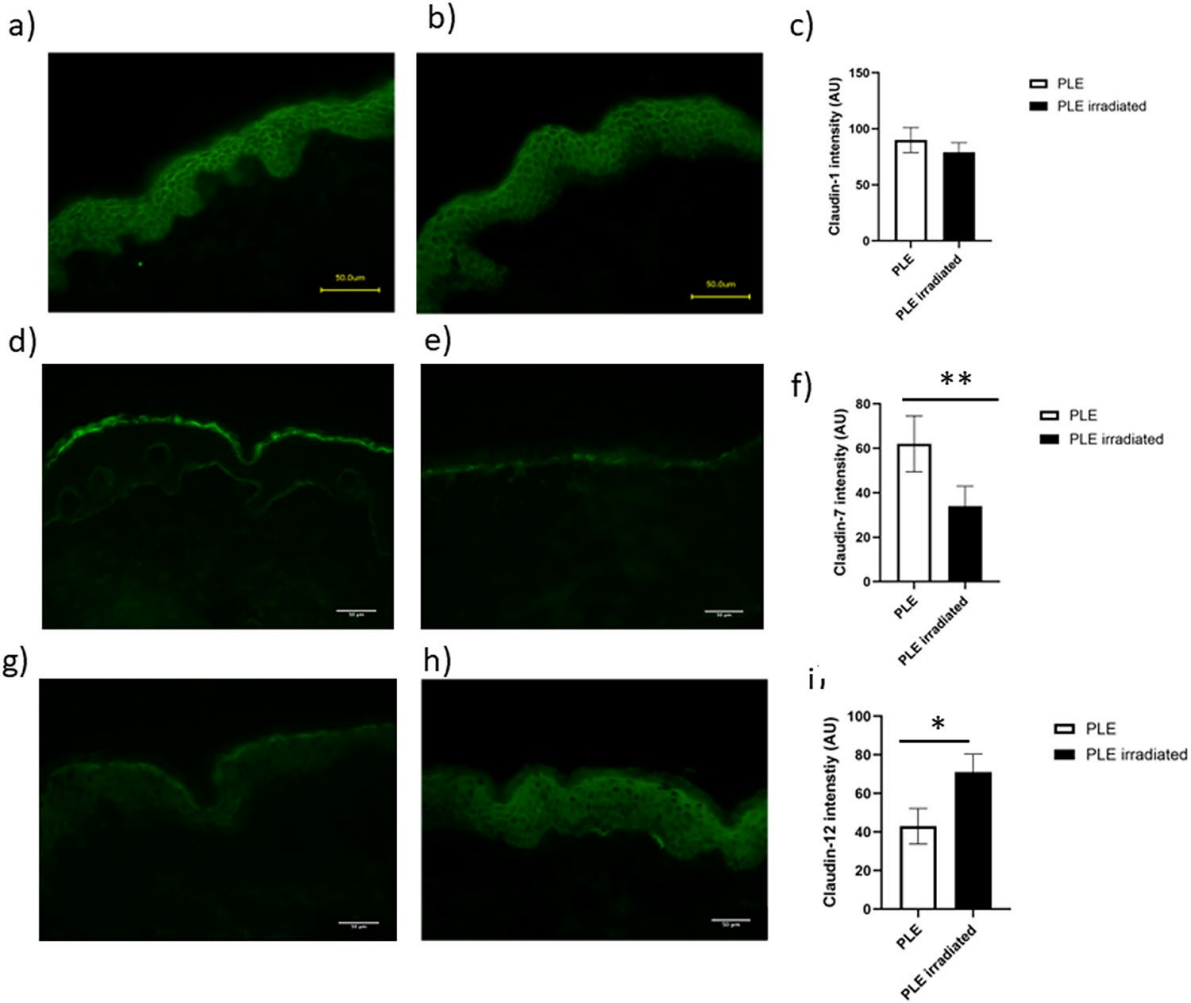
Irradiation results in alterations to claudins 7 and 12 in the skin of patients with PLE. Patients with PLE were subjected to a single dose of UVR and a biopsy taken 24 h post irradiation. The intensity of claudin-1 staining (**a**-**c**) was unchanged. However, claudin 7(**d**, **e**, **f**) staining was significantly decreased by irradiation (*P* < 0.01, *n* = 7) while claudin-12 (**g**, **h**, **i**) staining was significantly increased (*n* = 7, *p* < 0.05). Data shown are mean ± SD

We next investigated the possible effects of UVR on barrier protein expression. A dose-series of UVR was given and 4 mm punch biopsies were taken from skin treated with the highest dose, 80 mJ/cm^2^, 24 h post UVR. No PLE lesions were provoked in PLE patients. Non-lesional, UVR-exposed PLE patient skin was thus compared with UVR-exposed healthy control skin. In healthy skin, UVR exposure resulted in a significant decrease in claudin-1 expression (*p* < 0.01, Fig. 4a-c), while in contrast, claudin-1 expression in UVR-exposed PLE patient skin was identical to that in unexposed PLE patient skin (Fig. 5a-c). Claudin-4 expression was unaffected by UVR in either healthy skin or that from patients with PLE (not shown). Claudin-7 expression was unaltered by UVR in healthy skin (Fig. 4d-f) but was significantly decreased by UVR in PLE patient skin (Fig. 5d-f). Claudin-12 expression was unchanged by UVR in healthy skin (Fig. 4g-i) but was increased in PLE (Fig. 5g-i). The expression of occludin, filaggrin, involucrin and loricrin was unaltered by UVR in both groups as was the expression of Ki-67 (data not shown).

### Trans-epidermal water loss

**Fig. 6:**
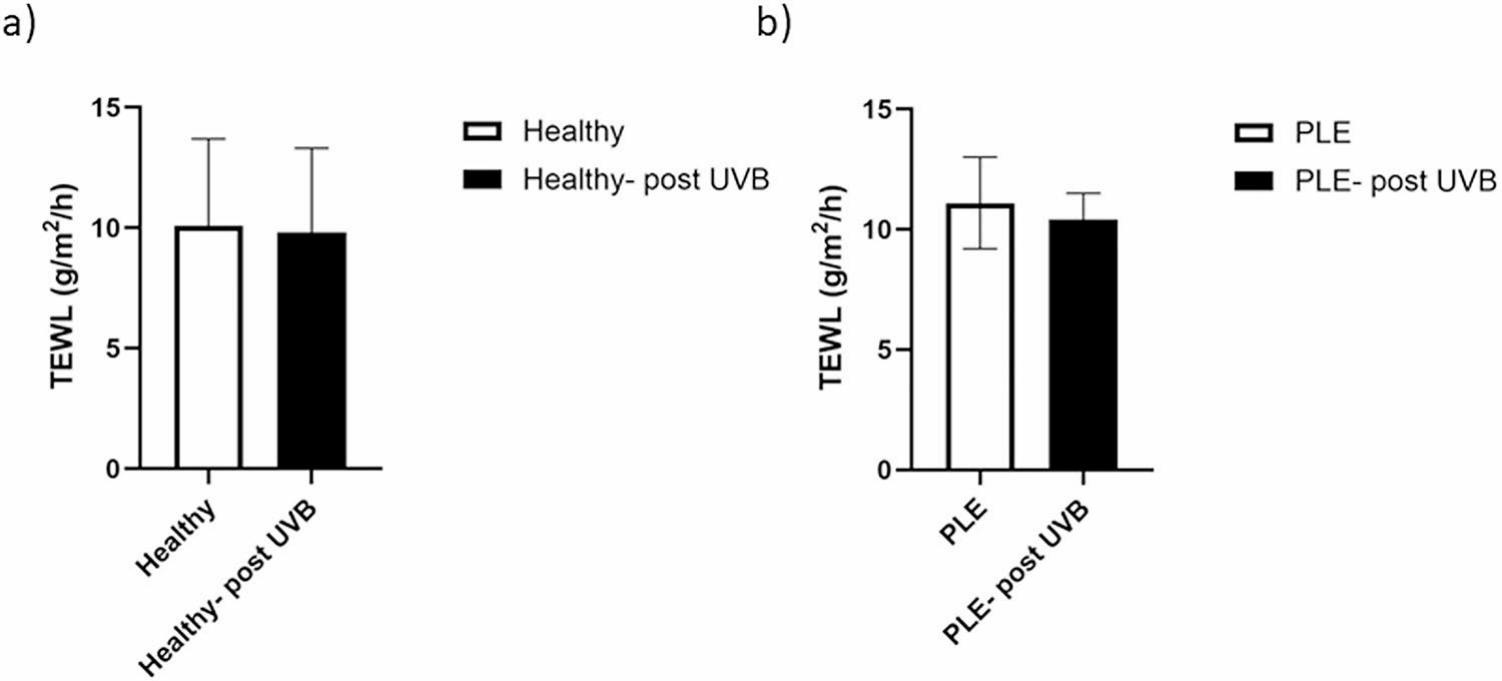
Trans-epidermal water loss is the same in healthy skin versus skin from PLE patients. Trans-epidermal water loss in healthy volunteers (**a**) vs. patients with PLE (**b**). There was no difference between the two groups. Furthermore, UVR-exposure did not result in an increase in TEWL at 24 h post irradiation. Data shown are mean ± SD

Under these experimental conditions, TEWL was identical between healthy volunteers and patients with PLE at baseline. No influence of the single UVR dose was detected in either group (Fig. 6a, b).

## Discussion

This original study investigated barrier protein expression and function in patients with the common photodermatosis, PLE. Our study provides novel findings on baseline differences in TJ structure and Ki-67 expression in the unexposed, non-inflammed skin of healthy volunteers compared with PLE patients, and shows early differences in their UVR responses. The principal finding of this study is that the TJ protein claudin-1 is significantly reduced in the unexposed skin of PLE patients compared with that of healthy volunteers (Fig. 1), and additionally differential responses to UVR exposure are seen in further TJ proteins claudin-7 and claudin-12 (Fig. 5).

Unlike other claudins, claudin-1 is ubiquitously expressed in epithelia [26]. Thus, claudin-1 is considered to be the ‘back-bone’ of the TJ [26]. In skin, complete loss of its expression by genetic ablation results in mice that die of dehydration 24 h after birth, associated with excessive TEWL [16]. We found no difference in TEWL between healthy volunteers and patients with PLE, where claudin-1 was reduced but not lost entirely. This is in agreement with Bergman et al. [27] who also noted that TEWL was quite stable for a wide range of claudin-1 expression. Furthermore, Arnold et al. [28] have recently demonstrated that complete ablation of claudin-1 in keratinocytes still results in their ability to form a TJ barrier, suggestive of the idea that other TJ proteins may be able to compensate to some degree. TEWL is a measure of whole barrier function, taking into account all layers of the skin including the *stratum corneum.* Changes in TEWL have been noted in PLE lesions [29], and it is possible that in non-lesional skin of PLE patients, as examined in the current study, an abnormality of TJ barrier occurs without any change in water loss from the skin, i.e. with sufficient protein retained to maintain TEWL. Furthermore, since the precise roles of claudins in skin have yet to be established, with most research relating to their role in simple epithelia such as in the gastrointestinal tract [30], it is possible that claudin-1 in the human epidermis may be playing other key roles.

In human skin, reduced expression of claudin-1 is associated with atopic dermatitis, where the barrier of skin is known to be compromised. However, in some patients, atopic dermatitis is also associated with deficiency of filaggrin, a major protein of the stratum corneum [31]. In PLE patient skin, we did not demonstrate any change in filaggrin expression thus suggesting that the deficiency of the barrier is fundamentally different in these two conditions. The loss of claudin-1 occurred across the whole epidermis but in some samples appeared to be greatest in the basal layer. Among TJ proteins, claudin-1 is unique in its expression throughout all epidermal layers. Since the TJ barrier is located in the granular layer, this supports that claudin-1 may have other functions alongside its role as a TJ barrier protein. Indeed, recent work suggests that the requirement for claudin-1 in wound healing is a non-barrier effect of this protein [32]. Our observation of an increase in the number of cells expressing Ki-67 in PLE is in keeping with studies suggestive of a link between cell proliferation and loss of claudin-1. In keratinocytes, knock-down of claudin-1 is known to increase keratinocyte proliferation [17, 28]. These data also point to roles for claudin-1 beyond one of barrier function.

The current study additionally provides new insights into the impact of UVR exposure on healthy human skin. While UVR is known to influence the skin barrier [33], no previous human in vivo studies have been reported on its impact on epidermal TJs. UVR-exposure of the skin of healthy volunteers resulted in loss of claudin-1. This is supported by the findings of Yuki et al. who also demonstrated UVR-induced loss of the TJ barrier in human skin xenografted onto mice [22]. Loss of claudin-1 has also been observed in the skin of patients with another photoimmune disorder, chronic actinic dermatitis (CAD) [34]. Investigation of the potential mechanisms provided three notable findings. Firstly, the skin of patients with CAD demonstrated overexpression of a micro RNA (hsa-miR-31-3p), secondly hsa-miR-31-3p was overexpressed on skin exposure to UVR, and thirdly over-expression of hsa-miR-31-3p in vitro resulted in reduced claudin-1 expression [34]. Overall, these findings provide a potential mechanistic link between UVR and reduced claudin-1 expression in CAD and suggest that exploration of hsa-miR-31-3p is worthy of future study in the context of PLE.

In the present study, UVR exposure resulted in no further loss of claudin-1 in the skin of patients with PLE but did induce changes to other TJ proteins. In healthy volunteers, there was a trend towards increased claudin-7 expression in response to UVR although this did not reach statistical significance. In contrast, in PLE patients, expression of claudin-7 was significantly reduced while claudin-12 was significantly increased at 24 h post-UVR. Both of these claudins are associated with ion transport pathways in other epithelia; their role in skin is as yet poorly defined and our findings highlight the need for their further study [26].

Whilst the precise implication of loss of claudin-1 in PLE skin is unclear, we hypothesise that the observed disruption of the TJ barrier may enhance penetration of the putative photoantigen within the skin. Whether the photoantigen is exogenous or endogenous, TJ dysfunction could allow access to relevant components of the immune system [35], ultimately resulting in the delayed hypersensitivity response observed in PLE. Furthermore, interdependence between TJs and the ability of Langerhan’s cells to survey and present antigens may enhance putative photoantigen presentation and T cell activation [36]. Loss of claudin-1 may also be related to the failure of the apoptotic process which is evident in PLE [37]. Studies, mostly in cancer cell types, have shown associations between claudin-1 expression levels and apoptosis but the data are mixed. In breast cancer cells, where claudin-1 levels are reduced, re-introduction of claudin-1 triggers apoptosis [38], while other studies suggest that increased claudin-1 expression contributes to an anti-apoptotic effect [39]. Further studies may examine claudin-1 mRNA to assess whether loss is transcriptional or solely related to loss of the protein. It would also be intriguing to examine how our novel findings are affected by a course of phototherapy. Our observation that the major TJ protein claudin-1 is depleted in the skin of PLE patients suggests a possible role for barrier dysfunction in PLE, which has been hitherto overlooked.

## Conclusion

This study has made the novel discovery that tight junction protein expression is aberrant in people with PLE, both in their skin at baseline and in their skin’s response to UVR exposure. This indicates a potential role for tight junction proteins in PLE pathogenesis, and warrants further study.

## Data Availability

Data is provided within the manuscript.
